# Natural Products and Health

**DOI:** 10.3390/nu16030415

**Published:** 2024-01-31

**Authors:** Joanna Bartkowiak-Wieczorek, Edyta Mądry

**Affiliations:** Physiology Department, Poznan University of Medical Sciences, 6, Święcickiego Street, 60-781 Poznan, Poland; emadry@ump.edu.pl

A natural product is an organic compound from a living organism that can be isolated from natural sources or synthesized.

This paper aims to highlight the scientific achievements published in the Special Issue of *Nutrients* “Natural Products and Health”, which comprises nine original papers and three reviews. The review articles focus on the Acai Palm, saponins found in allium vegetables, and dietary recommendations for individuals with inflammatory bowel disease. The original papers cover topics such as olive oil, royal jelly, hemp, Paeonia seed oil, Boswellia serrata resin, phloretamide (a flavonoid present in apple juice), catalpol (an iridoid from Rehmannia glutinous roots), and a flavone found in the leaves of Crocus species.

**The açaí palm** (*Euterpe oleracea*) and its berries, which contain a high proportion of fats (50% of their composition), are well-known for their potent antioxidant and anti-inflammatory properties [1,2]. Açaí is rich in amino acids, polyphenols, lignan isolates, different fatty acids, and sterols, and effectively counters oxidative stress and regulates pro-inflammatory genes (TNF-α, COX-2, NF-κβ) in vitro [3]. Açaí exhibits analgesic and anti-inflammatory effects, and its oil (EOO) serves as an effective antibacterial agent against *Escherichia coli*, *Enterococcus faecalis*, *Pseudomonas aeruginosa*, and *Streptomyces aureus* [4,5,6]. Moreover, it shows promise in inhibiting cancer cell growth [7,8], improving heart function, reducing blood pressure, and enhancing vascular health [9,10]. Açaí may also provide protection to the kidneys, reducing damage and enhancing their function, and could combat neurodegenerative diseases through antioxidant and anti-inflammatory mechanisms and brain protein homeostasis. Additionally, it demonstrates potential antidiabetic, antidyslipidemic, and hepato- and nephroprotective benefits. Clinical trials have validated açaí’s effectiveness in alleviating prostate cancer, mitigating risk factors associated with metabolic syndrome, and addressing auditory dysfunctions [11]. 

**Allium** contains steroidal saponins that exhibit a range of diverse biological activities [12,13,14], including hypoglycemic activity, potentially regulated by visfatin, and antiplatelet activity that reduces ADP-induced aggregation. Additionally, saponins exhibit gastroprotective effects, bolster the immune response, and demonstrate anti-inflammatory properties by restraining NO production by lipopolysaccharide (LPS). Saponins also demonstrate cytotoxicity and antitumor effects against various tumor cell types, possess antifungal and antibacterial properties, inhibit specific enzymes (Na, K-ATPase, cAMP phosphodiesterases), display antispasmodic activity, influence calcium ion regulation in cardiomyocytes, and exhibit neuroprotective effects [15,16,17]. In a comprehensive literature review, Wang et al. emphasized the significant role of steroidal saponins and elucidated their biosynthetic process. They determined the biosynthetic pathways of several pivotal compounds, contributing to a deeper understanding of this field [18].

The impact of phenolic compounds found in **extra virgin olive oil (EVOO)**, namely hydroxytyrosol (htyr), oleocanthal (ole), and tyrosol (tyr), was examined in cultured human fibroblasts (specifically, the human skin fibroblast cell line CCD-1064S) [19,20]. These compounds significantly increased both the proliferation and migration of fibroblasts, with oleocanthal demonstrating the most pronounced effect at concentrations of 10^−6^ M and 10^−7^ M. The treatments also resulted in a noteworthy elevation in fibronectin and α-actin expression in fibroblasts. Notably, no significant alterations were observed in cell cycle distribution or DNA integrity, suggesting the safety of these compounds. These findings underscore the potential of EVOO polyphenols in promoting tissue repair and regeneration, suggesting potential applications in wound healing processes such as cell adhesion, chemotaxis, and phagocytosis [21,22].

**Royal jelly (RJ)** has been employed for treating non-alcoholic fatty liver disease. The application of RJ resulted in reduced weight gain, alleviated hyperinsulinemia, and improved glucose tolerance. It also lowered liver enzymes and leptin, restored adiponectin levels, and decreased inflammatory markers (IL-6, TNF-α) [23,24,25]. In rats fed a high-fat diet, RJ enhanced their lipid profiles. Furthermore, RJ treatment led to the restoration of AMPK activation and the expression of genes responsible for fat metabolism, such as SREBP1 and PPARα. Histological examination revealed improved liver structure with fewer fat vacuoles in RJ-treated rats. However, when RJ was co-administered with an AMPK inhibitor, compound C (CC), these beneficial effects were attenuated [26].

**Suffruticosol C**, derived from the seeds of Paeonia species [27,28], exhibits significant antitumor effects against various cancer cell lines (Caco2, H1299, HCT116, HepG2, and PC3 cells). It induces cell death, autophagy, and cell cycle arrest by inhibiting the mTORC1 pathway [29,30,31,32]. Suffruticosol C significantly suppresses the growth of different lines of cancer cells and demonstrates more potency than resveratrol. It promotes autophagy by increasing autophagy markers, up-regulating the expression of genes associated with lysosome biogenesis, and starting the autophagosomal process. Suffruticosol C suppresses cell proliferation and division and, in a dose-dependent manner, inhibits mTORC1 activation, suggesting its potential as an anti-cancer agent that targets autophagy and cell cycle-related mechanisms [33,34,35,36,37,38,39,40,41,42,43,44,45,46,47].

**6-Hydroxyflavanone (6-HF)** displays anti-inflammatory, antioxidant, and anti-neuropathic effects [48,49,50]. It induces cell death, autophagy, and cell cycle arrest in a dose-dependent manner, suggesting its potential as an anticancer agent. In silico docking investigations and in vitro studies revealed that 6-HF has essential binding interactions with the catalytic site residues of the COX-2 enzyme, demonstrating significant inhibitory activity against COX-2 and 5-LOX enzymes. In vivo studies demonstrated that 6-HF highly prolonged the latency of responses in mice, indicating its thermal anti-nociceptive effects. Additionally, it displayed anti-inflammatory activity in a carrageenan-induced paw edema test and effectively alleviated allodynia and vulvodynia, both static and dynamic, in a diabetic neuropathy model induced by streptozotocin (STZ). These findings imply that 6-HF potentially has anticancer properties, specifically targeting autophagy and cell cycle-related mechanisms [51,52].

**11-keto-β-boswellic acid (AKBA)**, a key constituent of the natural resin Boswellia serrata, exhibits antioxidant properties by stimulating the Nrf2/antioxidant axis and possesses anti-inflammatory properties, targeting NF-κB p65, IL-6, and TNF-α [53,54,55]. In diabetic rats, it elevated insulin levels, aiding in blood glucose regulation. AKBA also lowered high blood sugar levels and reduced lipid accumulation in the heart and liver of rats. It stimulated glucose uptake and oxidation and enhanced fatty acid oxidation in the heart, leading to normalized cardiac metabolism. Furthermore, it bolstered the Nrf2 antioxidant pathway, thereby reducing oxidative stress [55,56,57,58,59]. AKBA suppressed NF-κB and inflammatory cytokines, resulting in reduced inflammation. It played a crucial role in AMPK activation, preserving cardiac structure and function, improving glucose and lipid profiles, and modulating cardiac metabolism in diabetic rats. When AMPK was inhibited, these benefits were reversed, underscoring the significance of AKBA’s role in AMPK regulation [60].

**Phloretamide**, a derivative of phloretic acid found in apple juice [61,62,63], exhibited promising potential in mitigating non-alcoholic fatty liver disease (NAFLD) in streptozotocin (STZ)-induced diabetic rats. This compound significantly increased the rats’ body weight and improved glucose regulation, with notable reductions in fasting glucose and hepatic levels of, G-6-Pase, IL-6, FBP-1, NF-κB, MDA, TNF-α. It simultaneously raised levels of CAT, GSH, HO-1, and SOD and hepatic levels of hexokinase and glycogen and positively influenced pancreatic structure by increasing islet size and cell count. Additionally, it favorably impacted lipid profiles by reducing serum levels of FFAs and rectifying lipid imbalances. Phloretamide effectively reversed adverse changes in oxidative stress and inflammation markers. It significantly impacted the hepatic Keap-1/Nrf2 axis, with more pronounced effects at higher doses. Histological improvements were observed in liver tissues from STZ-induced diabetic rats treated with phloretamide, particularly at a dose of 200 mg/kg, showing nearly normal hepatocytes and reduced cytoplasmic fat deposits [64].

**Catalpol** (CAT), an iridoid glucoside derived from the root of Rehmannia glutinosa [65], demonstrates nephroprotective effects in murine models of chronic kidney disease (CKD). CAT effectively mitigates adenine-induced alterations in the body, water intake, urine volume, and plasma concentrations of creatinine and urea [66,67,68,69,70,71,72,73]. Moreover, CAT reduces adenine-induced kidney injury by lowering levels of kidney injury molecule-1, adiponectin, cystatin C, and neutrophil gelatinase-associated lipocalin. In an adenine-treated group, CAT pre-treatment significantly reduced inflammation and oxidative stress markers (TNFα and IL-6, NF-κB). Histologically, CAT demonstrates notable effects in reducing tubular necrosis, interstitial fibrosis, and dilation in the kidney. The beneficial effects of CAT against adenine-induced CKD in mouse models involve mechanisms such as sirtuin-1 activation and NF-κB inhibition [74].

**Cannabidiol** (CBD) and tetrahydrocannabinol (THC) are the principal components of Cannabis sativa [75]. In the Special Issue “Natural Products and Health”, two papers concern Cannabis sativa. In the first paper, hemp extract prepared from plants with reduced THC content was orally administered to rats. THC is responsible for the hallucinogenic and euphoric effects of hemp preparations, and its reduction in hemp preparations used by humans is desirable. In oral hemp treatment, the pharmacokinetics and bioavailability of CBD and THC are significantly influenced by the solvent. The authors showed that for hemp extract dissolved in rapeseed oil, the total bioavailability of CBD and THC was higher than for Cremophor. Notably, higher CBD concentrations than THC were observed in the whole blood and the brain. However, some CBD underwent conversion into THC within the body, a factor to be considered when using Cannabis sativa for medicinal purposes in humans [76].

The authors of the second paper on hemp examined how CBD influenced the development of alcohol addiction in a rat model. The sedative and hypothermic effects of alcohol increased with the elevation of blood alcohol concentration [77]. Given that tolerance is considered to be a precursor to drug addiction, it is suggested that CBD can impede the development of alcohol dependence. On the molecular level, the most expressed effect of the ethanol–CBD intervention was observed in the striatum, where CBD inverted the ethanol-induced down-regulation of CB2R gene transcription. The opposite effect was observed for the mRNA of CB1 and dopaminergic receptors (DRD1, DRD2) [78].

**Inflammatory bowel disease (IBD)** is characterised by intestinal inflammation resulting from both genetic and environmental factors, among which diet plays the most critical role. Through a critical analysis of data on the use of selected diets (Low-FODMAP diet, Exclusive Enteral Nutrition, Specific Carbohydrate Diet, Anti-Inflammatory Diet) and based on available medical data, the authors prepared guidelines for patients and clinicians regarding best practices in diet modification for treating IBD. They concluded that a diet high in selected fats, artificial sweeteners, carbohydrates, and some additives, specifically carrageenan, exacerbates IBD, just as a diet rich in meat also has an undesirable effect on the course of IBD. In contrast, dietary fiber, fruits, omega-3 fatty acids, and Curcumin, a turmeric component, are considered protective in IBD management [79].

The search for new human-health-promoting molecules of natural origin is a topic that has attracted much interest. Natural products have been used for medicinal purposes for thousands of years and are still a vital, available, and cheap source of substances of pharmacological value [80].

They are also integral in the field of functional food and can contribute to improving the health-promoting properties of food matrices. This is especially important in the case of lifestyle-affecting diseases, such as inflammatory bowel disease (IBD) or fatty liver disease, in terms of both treatment and prevention. As mentioned above, components like dietary fiber, omega-3 fatty acids, and curcumin show protective effects in managing IBD, and phloretamide, found in apple juice, may support the treatment of liver disorders, including steatosis.

The importance of this knowledge lies in the fact that education and dietary impact can influence vast populations at a relatively low cost [80].

## Abbreviations

5-LOX	5-Lipoxygenase
6-HF	6-Hydroxyflavanone
AKBA	11-keto-β-boswellic acid
AMPK	AMP-activated protein kinase
CAT	catalpol
CBD	cannabidiol
CBR	cannabinoid receptor
COX-2	cyclooxygenase 2
DM	diabetes mellitus
DNA	deoxyribonucleic acid
DRD	dopamine receptor
EVOO	extra virgin olive oil
FFA	free fatty acid
Htyr	hydroxytyrosol
IL-6	interleukin 6
mRNA	messenger ribonucleic acid
mTORC1	mammalian target of rapamycin complex 1
NF-κB	nuclear factor kappa B
Nrf2	nuclear factor erythroid 2
Ole	oleocanthal
RJ	royal jelly
SREBP1	sterol regulatory element-binding protein 1
STZ	streptozotocin
THC	tetrahydrocannabinol
TNF-α	tumor necrosis factor α
Tyr	tyrosol
